# Practice patterns, experiences, and challenges of German oncology health care staff with smoking cessation in patients with cancer: a cross-sectional survey study

**DOI:** 10.1007/s11764-023-01501-2

**Published:** 2023-11-28

**Authors:** Frederike Bokemeyer, Lisa Lebherz, Carsten Bokemeyer, Jeroen W. G. Derksen, Holger Schulz, Christiane Bleich

**Affiliations:** 1 Department of Medical Psychology, University Medical Center Hamburg Eppendorf, Martinistraße 52, 20246 Hamburg, Germany; 2 Center for Oncology, II. Medical Clinic and Polyclinic, University Medical Center Hamburg Eppendorf, Martinistraße 52, 20246 Hamburg, Germany; 3 Division Julius Center for Health Sciences and Primary Care, Department of Epidemiology and Health Economics, University Medical Center Utrecht, Utrecht University, Heidelberglaan 100, 3584 CX Utrecht, The Netherlands

**Keywords:** Cancer, Health staff, Smoking cessation, Smoking relapse, Psycho-oncology, Health service research

## Abstract

**Purpose:**

Often, cancer patients do not receive education about the negative consequences of smoking on the treatment outcome. To support cancer patients in the process of smoking cessation, it is essential to involve oncology staff. This study aims to learn about the experiences and attitudes from the point of view of oncology staff and, thus, how a smoking intervention should be designed. The study aims to engage all oncology staff due to the unclear responsibility for providing smoking cessation education, support, and motivating cancer patients to quit smoking.

**Methods:**

*N* = 354 German oncology staff (oncologists, nurses, psycho-oncologists, others) filled out a 5-point Likert scale–based questionnaire regarding practices, potential barriers, and attitudes towards smoking cessation between October 2021 and June 2022. The questionnaire was developed by Derksen et al. (2020), translated and slightly modified for the use of this study. It was distributed to all leading oncology staff in our Cancer Center Network with a request to share with all oncology staff. Flyers were also handed out in all oncology wards and outpatient clinics in the same Cancer Center Network.

**Results:**

Most oncology staff ask cancer patients about their current smoking status (curative, *M* = 2.27; SD = 1.59; palliative, *M* = 2.90; SD = 1.83), but they rarely treat or refer patients for a smoking cessation intervention (curative, *M* = 4.78; SD = 1.20; palliative, *M* = 4.99; SD = 1.06). Smoking behavior of curative cancer patients is addressed more than that of palliative cancer patients (*d* =  − 37). Regression analyses of key dependent variables showed that profession, setting, and the belief that continued smoking affects treatment outcome explained the variance of asking patients if they smoke, advising to stop smoking and lack of time (without profession).

**Conclusion:**

Involving oncology staff in motivating cancer patients who smoke to quit and referring them to smoking cessation services should take the different attitudes and knowledge of the staff into account to improve treatment that supports tobacco cessation.

**Implications for Cancer Survivors:**

Cancer patients have special needs when it comes to a cessation program. In the long term, survivors will benefit from tailored smoking cessation education and services provided by oncology staff to help them quit smoking after a cancer diagnosis.

**Supplementary Information:**

The online version contains supplementary material available at 10.1007/s11764-023-01501-2.

## Background

With a general prevalence of smoking in Germany of approximately 24% for women and 34% for men, smoking remains a major health problem in Germany and is one of the leading causes of premature death [1]. However, especially continued smoking after a cancer diagnosis can be associated with poor clinical outcomes. Available studies show that up to 60% of cancer patients continue to smoke after their cancer diagnosis [2, 3]. Consequences include an increased risk of side effects [4], worsened wound-healing [5], reduced effectiveness of systemic or radiotherapy [6, 7], and an increased risk of second primary tumors [7] or recurrences [8]. In addition, long-term survival is reduced in cancer patients who continue smoking as compared to patients who quit smoking after diagnosis or have never smoked [9, 10]. Therefore, it is important to inform all patients with cancer about the health consequences of (continued) smoking, especially its negative effects on cancer treatment, and subsequently to motivate them to stop smoking [11]. A recent randomized controlled trial on smoking cessation in cancer patients showed that long-term smoking cessation advice increased the likelihood of smoking abstinence compared with short-term advice [12].

However, previous studies have shown that up to 60% of patients with cancer are not asked about their current smoking status and thus do not get informed about the consequences of continuing smoking after the cancer diagnosis [3, 13]. Recently, Derksen et al. (2020) performed a survey study among European oncologists to study practices patterns regarding smoking cessation after a cancer diagnosis, with a particular focus on comparing curative and palliative settings. Their study included 544 oncology physicians from 16 European countries and showed that oncologists were more likely to address tobacco use in the curative setting compared to the palliative setting but discussed medication options and/or offered smoking cessation support only in a minority of cases. Further, it was also reported that the discomfort of asking patients to quit the popular habit of smoking and doubt that smoking has a strong impact on treatment were major barriers for physicians to recommend smoking cessation, especially in the palliative setting [14]. In both settings, lack of time, resources, and training on how to provide smoking cessation support as well as patient resistance were reported by oncology physicians as the most common barriers. These findings are comparable to other studies that investigated potential barriers to providing smoking cessation support in patients with cancer [15, 16]. Yet, oncological health staff in particular could have a major impact on the patients’ attitudes towards smoking and smoking behavior and could even serve as role models [17]. They may set an example of healthy behavior, e.g., by abstaining from smoking in front of their patients.

In order to integrate appropriate smoking cessation interventions into routine oncology care, it seems important to consider not only the perspectives, experiences, and opinions of oncologists, but also those of other staff involved in oncology care and cancer treatment, such as nurses, psycho-oncologists, and social workers. In addition, in order to provide the right type of intervention to the right patients, it is of interest to identify factors related to oncology staff’s beliefs or behaviors regarding smoking cessation in cancer patients.

To the best of our knowledge, associations between these beliefs or behaviors with sociodemographic or occupational factors have hardly been investigated. It is important to better understand whether sociodemographic factors (e.g., gender, age), but also the medical profession (e.g., doctor or nurse), the place of work, the type of cancer entity, or staff’s own smoking status, are related to their behavior towards actively smoking patients. Relevant factors to be considered have previously been identified by Derksen et al. (2020) [14], which will be used and further explored in the current study. Therefore, our aims were to (i) survey different oncology healthcare staff in a German comprehensive cancer center network about their attitudes and experiences (e.g., interaction with cancer patients, perceptions of continued smoking after a cancer diagnosis, barriers to helping cancer patients quit smoking), (ii) exploratively identify factors associated with different approaches to dealing with smoking in cancer patients by health care staff, and (iii) to find out how oncology staff can support their smoking patients to quit smoking as soon as they are diagnosed. The focus of this study is to involve all oncology staff, as there are no established roles for who should address the issue of smoking cessation in cancer patients due to the lack of clear responsibility for who should primarily address smoking cessation education and support and who should primarily motivate cancer patients to stop smoking.

## Methods

### Design

This cross-sectional study surveys the smoking cessation practice patterns, perceptions on barriers, and attitudes of oncology health staff involved in the treatment of patients with cancer. This study will provide information on whom to address and what issues are mostly relevant to consider when developing a comprehensive smoking cessation program.

### Recruitment and procedure

All German-speaking oncology health care staff within the network of one major comprehensive cancer center in Hamburg, Germany, who routinely work with cancer patients and are at least 18 years old were eligible to participate. The study was conducted as an online survey (LimeSurvey, server of the University of Hamburg); a paper–pencil version was provided upon request. All leading oncology staff within the network of the University Cancer Center Hamburg were contacted by email including a brief description of the study and a link plus QR (quick response) code to access the web-based survey. Participants were encouraged to share the survey link with colleagues and staff. After 3 weeks, a reminder email was sent to all selected individuals. The leading oncological staff was contacted in the same way and asked to advertise participation in the study. Unfortunately, it cannot be verified to what extent this was followed. Flyers were also distributed to oncological wards, outpatient clinics and inpatient clinics (specifically head and neck, gynecology, lung, prostate cancer, and general cancer units) in the catchment area of our research group. Participation in this study was anonymous, voluntary, and without any financial or other incentives. All participants provided informed consent before starting the survey.

### Measurements

A questionnaire developed by Derksen et al. (2020) [14] based on the American Society of Clinical Oncology survey [18] was slightly modified for use in this study. The 53-item questionnaire was translated from English into German and slightly culturally adapted for, e.g., staff training and titles. The main extension of the original study by Derksen et al. (2020) is the inclusion of all health care staff and not only physicians as the target group. This was considered for all items, and therefore, in some cases, response options and questions were added, e.g., what is your profession? For anonymity reasons, the response option for the age question was changed from a free text option to three different response options (< 40 years, 40–49 years, ≥ 50 years). We also added a question “I ask my patients if they have smoked in the past” to collect information on past smoking status of patients. Also, two question were added to assess (i) the smoking history of the healthcare staff in our study (never smoked (less than 100 cigarettes) or more than 100 cigarettes in their lifetime), and (ii) current smoking behavior (current non-smoker, daily smoker, smoke several times a week).

The questionnaire covered three different main topics, i.e., communication with patients, healthcare staffs’ perception of continued smoking after a cancer diagnosis, and barriers to supporting cancer patients in smoking cessation. Information was collected for both the curative and the palliative settings. We followed the WHO definition of palliative care as the care of patients with life-threatening cancer, regardless of their specific cancer diagnosis [19].

Part I on *communication with patients* consisted of questions they asked their patients about their smoking habits at first contact and during follow-ups, the use of structured methods, motivation to quit smoking, counseling and offering medication options, and referral of patients to smoking cessation interventions as well as different approaches to patients with tobacco-related and non-tobacco-related cancers. The response options consist of a five-point Likert scale (always to never) and an option to indicate that the setting does not apply. The item “My interactions with patients regarding smoking/tobacco use differ between tobacco-related vs. non-tobacco-related cancers.” consists of the following 3 different response options: “no,” “yes, I discuss this mostly with patients with tobacco associated cancers,” “yes, I discuss this mostly with patients with non-tobacco associated cancers,” and again the option to indicate that the setting does not apply.

Part II on healthcare staffs’ *perception of continued smoking after a cancer diagnosis* included questions such as whether smoking could affect the outcome of treatment or whether smoking cessation should be a standard part of cancer treatment. It also contains questions on whether healthcare staff should be better trained to provide appropriate smoking cessation support, whether healthcare staff have been trained to provide smoking cessation support, what smoking cessation interventions, services, or treatment aids their workplace already provides, and who should ideally provide smoking cessation support. In contrast to the Derksen et al. (2020) [14] questionnaire, we used the following response options: I agree… “completely,” “mostly,” “somewhat,” “a little bit,” “not at all,” and an additional option to indicate that the setting does not apply. However, the following two items consisted of seven different response options: “Which of the following providers do you think is appropriate to provide cessation support for cancer patients on a regular basis?” and “What type of dedicated smoking/tobacco cessation program does your facility/practice have available for your cancer patients (check at least one).” Multiple responses are possible for both questions.

Lastly, part III on possible *barriers to supporting cancer patients in smoking cessation* to smoking cessation in cancer patients in both the palliative and the curative settings consisted of questions that covered both the patient side (e.g., costs) and the health staff side (e.g., lack of time, lack of experience, or lack of referral options). For response scaling, we used the same option as for the second part and also made the same changes to the original Derksen et al. (2020) [14] questionnaire.

In addition, the following characteristics of the respondents were asked: gender, age group, work setting, main staff tasks, own tobacco use. The original and the translated German questionnaire used in this study can be found in the supplements (S4 and S5).

A pilot test was conducted before the start of the study. Six oncology staff (one nurse, three oncology physicians, one psychosocial oncologist, and one psychosocial oncology researcher) completed the questionnaire and provided feedback. Based on the healthcare staffs’ feedback, mainly formal changes were made (e.g., layout changes to make the questionnaire easier to read). Finally, three questions from the original questionnaire were not included: academic title, staff experience with oncology patients in years, and working time with oncology patients in percent. The reason for this was a request to shorten the questionnaire to make it more attractive to many health staff, especially in the face of a stressful daily clinical work schedule.

### Data analysis

Data were collected online using LimeSurvey or by paper–pencil questionnaire upon request. Participants who completed less than 30% of the quantitative items were excluded. Under the assumption that missing values follow the missing at random principle, missing data were imputed using unbiased estimation (expectation–maximization algorithm). The variables used in the imputation model were all metric variables as well as the categorical items asking for respondent characteristics. Descriptive statistics using frequencies and means were calculated to describe participant characteristics in terms of demographics, medical activities, and own smoking status. Continuous data were summarized by mean, standard deviation (SD), and 95% confidence interval (95% CI).

Group comparisons between curative and palliative settings were made using general linear mixed models. Due to the more exploratory aim of the study, we did not correct the alpha level for multiple testing. To explain the variance of selected key items from each category, we analyzed eight potential predictors as fixed factors based on the literature and theoretical expectations, including gender (female, male, diverse), age group (≤ 40, 41–59, ≥ 59), occupation (physician, nurse, other), work setting (academic clinical setting, non-academic clinical setting), own smoking status (no, yes), proportion of tobacco-related tumor types in daily work, belief that smoking affects cancer treatments (five-point Likert scale), and clinical care setting (curative, palliative). Clinical care setting was included as a repeated measure. Tobacco association of the different tumors was used: The score was weighted based on the strength of the association. The higher the number of tobacco-associated tumors and the stronger the individual association of these with tobacco smoking, the higher the score.

We considered participants as a sample from a population and modeled them as a random effect, specifically including their random intercept. Results with *p* ≤ 0.05 were considered statistically significant. All data were analyzed using SPSS (Statistical Package for Social Sciences) version 27.0 (International Business Machines Corporation Crop) statistical software.

### Sample size and power

As a guide for the power analyses using PASS, Power Analysis & Sample Size version 16, we wanted to obtain a 95% CI for the five-point response scales that should be no greater than ± 0.20 around the respective means. A sample size of 297 respondents was needed to obtain a two-sided 95% CI with a distance from the mean to the limits of 0.20 when the estimated standard deviation is 1.75. Assuming a dropout rate of 30%, our goal was to enroll a minimum of 424 participants in the study.

## Results

### Participants

A total of 502 subjects were screened for eligibility of whom 61 clicked on the link, but never participated. Reasons for refusal to participate could not be determined. A total of 441 healthcare staff participated in the study, of whom 87 had to be excluded from the analyses (51 provided only basic parameters, 36 participants had more than 30% missing information). In the end, 354 oncology staff (oncologists, nurses, psycho-oncologists, others) who routinely work with cancer patients were surveyed between October 2021 and June 2022 and included in the analyses.

### Sample characteristics

Respondent sample characteristics are shown in Table 1. Half of the participants (50%) were under 40 years of age, 63% female, 55% worked as physicians, and mostly in a university hospital (69%). Lung tumors (36.2%), lymphomas (36.4%), and gastrointestinal tumors (31.6%) were the most frequently seen tumor types. Participants were also asked about their smoking status. The majority (84%) reported that they were currently non-smokers.

**Table 1:** Sample characteristics (*N* = 354)

	***N***	**%**
Age		
< 40 years	174	49.7
40–49 years	90	25.7
≥ 50 years	86	24.6
Gender		
Female	220	62.7
Male	129	36.8
Diverse	2	0.6
Profession		
Physician	194	55.3
Nurse	73	20.8
Psychologist	47	13.4
Other (dietitian, physiotherapist, surgical assistant, study nurse, social worker)	37	10.5
Specialty		
Medical oncology (systemic and medicinal tumor therapy)	176	50.1
Radiation therapy	10	2.8
Surgical oncology	60	17.1
Other	105	29.9
Workplace		
University hospital	244	69.3
Practice	58	16.5
Hospital	31	8.8
Other (medical supply center, medical institute)	19	5.4
Frequently treated cancer types (up to 3 entries)		
Lung cancer	128	36.2
Lymphoma	129	36.4
Gastrointestinal cancer	112	31.6
Breast cancer	77	21.8
Leukemia	78	22.0
Urogenital cancer	69	19.5
Head and neck cancer	67	18.9
Gynecologic cancer	44	12.4
Brain tumor	31	8.8
Skin cancer	14	4.0
Other	48	13.6
Smoking history		
Having smoked < 100 cigarettes	234	66.7
Having smoked > 100 cigarettes	117	33.3
Smoking status		
Currently non-smoker	297	83.9
Occasional smoker (sometimes)	33	9.4
Current smoker (every day)	20	5.7

Sums < 354 are due to missing values

### Communication with patients on smoking

**Fig. 1:**
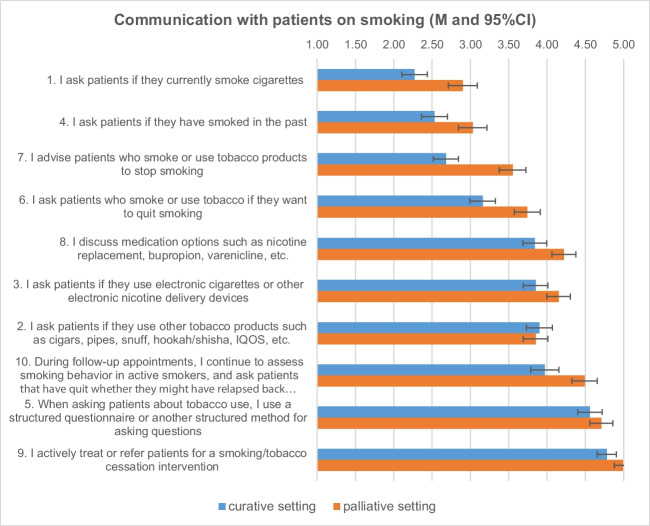
Interactions with patients concerning smoking (mean, 95% CI 1 = always, 2 = most of the time, 3 = some of the time, 4 = rarely, 5 = never)

This part included questions about communication and behavioral patterns used when working with cancer patients who smoke. The results are summarized in Fig. 1 and Table S1 supplements.

In both settings, higher frequencies were found for the two statements about the survey of current and past smoking status regarding cigarettes and for the two questions about the desire to stop smoking and the support offered to stop smoking. From the corresponding 95% CIs, it can be seen that for all four questions the scores are significantly lower, i.e., indicating a higher frequency of the corresponding action, in the curative than in the palliative setting. In addition, oncology staff were less likely to report asking about the use of other tobacco products (including e-cigarettes), re-addressing the topic of smoking at follow-up appointments, or discussing smoking cessation medication options, with significant lower frequencies of small effect size in the palliative setting. The lowest frequencies in this part are reported in both settings for the use of standardized survey instruments and for treatment or referral to treatment for smoking cessation.

For two predefined key items, i.e., “I ask patients if they currently smoke or use tobacco products.” and “I advise patients who smoke or use tobacco products to stop smoking,” we used multiple linear regression analyses to determine which of the a priori defined predictor variables were related to the two key variables (see Table 2). The results showed that being a physician, believing that active smoking or tobacco use interferes with treatment, and working in a curative setting were associated with a higher likelihood of asking patients if they smoke cigarettes. The belief that smoking affects treatment outcome was slightly positively associated with asking. Finally, participants were more likely to ask patients in a curative setting than in a palliative setting.

**Table 2:** Linear regression with the dependent variable “I ask patients if they currently smoke or use tobacco products” and “I advise patients who smoke or use tobacco products to stop smoking” (*N* = 339)

	Dependent variable: I ask patients if they smoke or use tobacco products^a^		Dependent variable: I advise patients who smoke or use tobacco products to stop smoking^a^	
Predictor variables	ß	CI [95%]	ß	CI [95%]
Gender (female vs. male^b^)	− 0.23	[− 0.57; 0.10]	0.02	[− 0.29; 0.34]
Age (reference category: > 59 years)				
≤ 40 years	0.24	[− 0.14; 0.63]	0.19	[− 0.17; 0.55]
41–59 years	− 0.02	[− 0.43; 0.39]	− 0.01	[− 0.39; 0.37]
Profession (reference category: other)				
Physician	− 1.63***	[− 2.03; − 1.24]	− 1.52***	[− 1.89; − 1.15]
Nurse	− 0.39	[− 0.83; 0.05]	− 0.42	[− 0.83; 0]
Work setting (academic healthcare vs. non-academic)	0.11	[− 0.22; 0.44]	0.31	[0; 0.61]
Smoking status (no vs. yes)	− 0.12	[− 0.54; 0.29]	− 0.40*	[− 0.79; − 0.01]
Proportion of tobacco associated tumor types in daily work	− 0.09	[− 0.23; 0.05]	0.12	[− 0.01; 0.25]
Believing smoking impacts treatment ^c^	0.20***	[0.10; 0.30]	0.39***	[0.29; 0.49]
Setting (curative vs. palliative)	− 0.40***	[− 0.54; − 0.27]	− 0.58***	[− 0.73; − 0.44]

*p < 0.05

**p < 0.01

***p < 0.001

^a^1 = always to 5 = never

^b^Diverse: *N* = 0

^c^1 = agree strongly to 5 = do not agree

Furthermore, the same set of a priori–defined predictors was used to estimate the variance in the second dependent variable “I advise patients who smoke or use tobacco products to stop smoking” (see Table 2). The analysis showed that being a physician, believing that smoking affects treatment outcomes not being a smoker, and working in a curative setting were associated with a higher likelihood of advising cancer patients who smoke to quit. Being a physician and working in a curative setting moderately increased the frequency of giving advice, while believing that smoking affects treatment outcome also increased the frequency of giving advice.

For two other pre-specified items in this topic, i.e., “I actively treat or refer patients for a smoking/tobacco cessation intervention” and “When asking about tobacco use, I use a structured questionnaire or other structured method for asking questions,” we omitted the calculation because these items were too skewed in their distribution.

### Perception of continued smoking after a cancer diagnosis

**Fig. 2:**
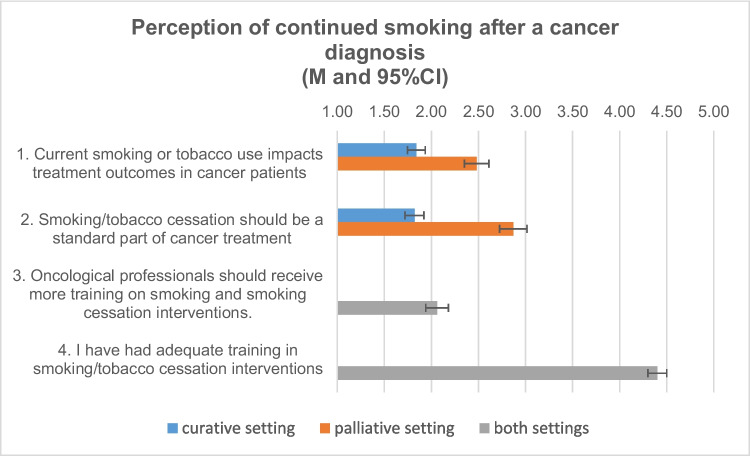
Perception of continued smoking after a cancer diagnosis (mean, 95% CI I agree 1 = completely, 2 = mostly, 3 = somewhat, 4 = a little bit, 5 = not at all)

The results of the topic “perception of continued smoking after a cancer diagnosis” are summarized in Table S2 and Fig. 2.

Regarding the perceptions of smoking continuation after cancer diagnosis (see Table S2), oncology staff were asked whether current smoking or tobacco use affects treatment outcomes in cancer patients. On average, oncology staff tended to mostly agree, with slightly higher agreement in the curative setting. Also, especially in the curative setting, oncology staff on average agreed that smoking or tobacco cessation should be a standard part of cancer treatment. For the palliative setting, oncology staff only moderately agreed. These differences are statistically significant with medium to large effect sizes.

When asked if oncology staff were adequately trained to provide smoking cessation interventions, oncology staff disagreed and agreed that more adequate training is needed.

In addition, oncology staff were asked to indicate which staff should be most likely to provide regular smoking cessation support to cancer patients. The most frequently mentioned staff were the primary care physician (63.59%), clinical support staff such as psychologists or social workers (50.25%), and the attending oncologist (42.31%). Less often mentioned staff were mid-level clinical staff such as nurse practitioners or physician assistant (30.77), MD level provider other than primary care physician (16.67), or others (7.69). Only a minority said that they would not use any of the above resources (2.05). In addition, oncology staff were asked to indicate what type of smoking cessation support is currently offered at the cancer center where they work. Most oncology staff (35.38%) indicated that they were not aware of any smoking cessation support services at their workplace, but some were aware of smoking cessation information materials (20.00%).

### Barriers to supporting cancer patients in smoking cessation

**Fig. 3:**
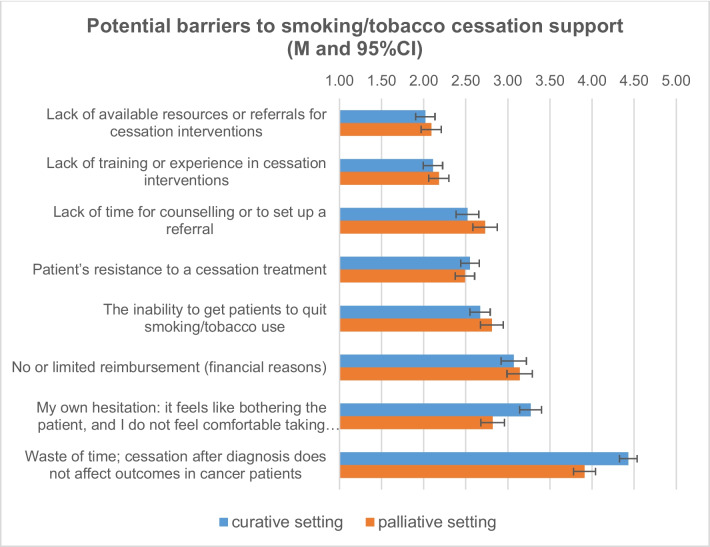
Potential barriers to smoking/tobacco cessation support (M, 95% CI I agree: 1 = completely, 2 = mostly, 3 = somewhat, 4 = a little bit, 5 = not at all)

Part 3 includes the results of all items related to potential barriers to smoking cessation for cancer patients perceived by oncology staff (see Table S3 and Fig. 3).

The items “Lack of available resources or referrals for cessation interventions.” and “Lack of training or experience in cessation interventions” were the most frequently agreed upon as potential barriers, with mean scores for these items corresponding to the “most” response category. The item “Lack of time for counseling or to set up a referral.” was also more frequently agreed upon as a potential barrier, with scores corresponding to the “most” and “somewhat” response categories. This was also true for the two items “Patient resistance to cessation treatment” and “The inability to get patients to quit smoking/tobacco use.” Two statements with the lowest overall approval differed significantly between the two settings: “My own hesitation; it feels like bothering the patient, and I do not feel comfortable taking something away they might enjoy doing.” and “Waste of time; cessation after diagnosis does not affect outcomes in cancer patients” show a higher agreement in the palliative care setting with small- to medium-effect sizes.

Table 3 shows the results of a multiple regression analysis explaining the variance of the dependent variable “Lack of time for counseling or set up a referral” as a barrier to smoking cessation in cancer patients.

**Table 3:** Linear regression with the dependent variable “Lack of time for counseling or to set up a referral” (*N* = 331)

Dependent variable: Lack of time for counseling or to set up a referral^a^		
Predictor variables	Estimates	CI [95%]
Gender (female vs. male^c^)	0.20	[− 0.10; 0.51]
Age (reference category: > 59 years)		
≤ 40 years	− 0.42	[− 0.77; − 0.07]
41–59 years	− 0.29	[− 0.66; 0.08]
Profession (reference category: other)		
Physician	− 0.45*	[− 0.82; − 0.09]
Nurse	− 0.33	[− 0.74; 0.08]
Work setting (academic healthcare vs. non-academic))	0.06	[− 0.24; 0.37]
Smoking status (no vs. yes)	0.16	[− 0.22; 0.53]
Proportion of tobacco associated tumor types in daily work	0.06	[− 0.07; 0.19]
Believing smoking impacts treatment ^b^	0.20***	[0.13; 0.28]
Setting (curative vs. palliative)	− 0.11*	[− 0.21; − 0.02]

*p < 0.05

**p < 0.01

***p < 0.001

^a^I agree 1 = completely to 5 = not at all

^b^Diverse: *N* = 0, hence gender was binary coded

^c^1 = agree strongly to 5 = do not agree

Three of the nine variables analyzed are significant: Working as a physician, believing that active smoking or tobacco use affects cancer treatment outcomes, and working in a curative cancer setting are associated with a higher likelihood of perceiving lack of time for counseling as a barrier to smoking cessation in cancer patients.

## Discussion

The aim of this cross-sectional study was to investigate how oncology staff view and deal with continued smoking in cancer patients. Complementing previous studies (Warren, 2013, Derksen, 2020), the study presented focused on the current attitudes and experiences of (1) the entire oncology staff, i.e., physicians, nurses, and psychologists, regarding smoking patterns and smoking cessation in oncology patients in a (2) large regional comprehensive cancer center network in Germany. The results of this study are also particularly important because (3) there is currently no structured smoking cessation program for cancer patients in Germany.

More than half of the respondents were oncology physicians, in addition to oncology nurses, psycho-oncologists, study nurses, or dietitians. The current smoking status of the participants was also assessed and showed that only a small proportion of staff were occasional or daily smokers. Compared to the German general population, with approximately one quarter of the population smoking, the overall percentage of smokers among oncology staff appears to be much lower. However, compared to the previous study by Derksen et al. (2020) [14], in which a total of 5% of European oncologists reported to currently smoke, the numbers are higher in our study population. A possible reason for this could be related to the composition of our sample. In addition to physicians, our study included other staff such as nurses, as being the second common participant in our study; psychologists; and other oncology support staff. Recent studies show that nurses are particularly at risk of becoming smokers, with a smoking prevalence of 19–40% [20].

### Communication with patients on smoking

Regarding most of the interactions staff had with cancer patients, the results showed that they were more open to interacting with patients treated with a curative intent about their smoking behavior than with cancer patients receiving palliative treatments. This result is not surprising. Smoking cessation interventions in palliative care are not accepted as standard practice [21]. Previous studies have shown that oncologists believe that patients’ stress and anxiety about treatment may increase if they try to quit smoking. In addition, oncologists are concerned that they may induce shame or guilt in patients by talking to them about the consequences of continuing to smoke [22]. This effect may be even more profound when caring for palliative care patients. These patients are facing the knowledge of their imminent death, and it is particularly difficult for treatment providers to deny them the pleasure of smoking. Nevertheless, it is important that oncology staff educate all cancer patients about the consequences of smoking and motivate them to quit, because even palliative care patients can benefit from smoking cessation by improving their quality of life [23].

However, even in the curative setting, staff reported almost never using a structured questionnaire or method, or rarely asking about smoked tobacco products other than cigarettes or e-cigarettes. It was also noticeable that in neither the curative nor the palliative setting was there a focus on motivating people to quit, encouraging them to quit onsite, or even referring them to a smoking intervention. The results also showed that staffs rarely discussed medical options to support smoking cessation, regardless of the clinical setting.

Regression analyses further showed that being a physician, believing in the impact of smoking on cancer treatment outcomes, and working in a curative setting increased the likelihood of asking cancer patients about their smoking status. These same factors plus, remarkably, not being a smoker were associated with a higher likelihood of actually advising cancer patients to quit smoking. These results suggest that palliative care physicians and other oncology staff working in both settings should be more involved in the process of screening and motivating patients to quit smoking. In this context, it is also important to educate staff who smoke themselves. For example, cancer patients spend most of their treatment time with nurses, and the proportion of smokers among nurses remains high [20]. Nevertheless, it is important to involve all oncology staff in the smoking cessation education and motivation process. They all need to understand the consequences of continued smoking and how best to motivate patients to quit. Patients may benefit from education and motivation from different types of oncology staff, e.g., from a medical perspective by physicians or from a psychological perspective by psycho-oncologists.

### Perception of continued smoking after a cancer diagnosis

Similar to oncologists’ perceptions of continued smoking after cancer diagnosis in the study by Derksen et al. (2020) [14], oncology staff in this study also agreed that smoking interferes with cancer treatment, and therefore, smoking cessation should be a standard part of cancer treatment, especially in a curative setting. All staff more or less agree that they have not been trained to provide adequate smoking cessation support and that they want to receive more training in the future. When asked who the primary provider of smoking cessation services should be, the most common response was, as also reported in the study by Derksen et al. (2020) [14], the primary care physician, followed by psychologists or social workers. One question is whether the primary care physicians themselves have sufficient knowledge and training to provide these services, or whether this is simply a shift in responsibility. The reported preference for the primary care physician as the main provider of smoking cessation services may also be due to a lack of familiarity among oncological staff about established smoking cessation services. There is a need for a closer collaboration between tobacco cessation program providers and oncology clinics. This would allow for targeted and direct referral of patients. Involving primary care physicians in this process could also be beneficial, as they often have a closer relationship with the patient and can act as an additional motivator.

A recent meta-analysis by Sheeran et al. (2019) analyzed the effectiveness of various current smoking cessation interventions for cancer patients. The included interventions had taken different approaches to who was responsible for providing smoking cessation services. This meta-analysis showed that smoking cessation support was most often provided by therapists/counselors, followed by nurses, and then by physicians or researchers [24]. A first step in oncology care could be to train oncology staff to educate smoking cancer patients about the consequences of smoking and to routinely refer them to smoking cessation services.

### Barriers to supporting cancer patients in smoking cessation

Another issue explored in this study was oncology staff’s perception of barriers to smoking cessation among cancer patients. The results showed that the staff identified their lack of training in providing smoking cessation support or education and the existing lack of available resources for referrals to smoking cessation interventions and patient resistance as major barriers to smoking cessation.

These results were very similar to those reported in the study by Derksen et al. (2020) [14]: European oncologists most frequently named the inability to get patients to quit, the patient’s resistance, the lack of time for counseling, and a lack of training as major barriers.

We further analyzed the association between several personal and sociodemographic factors and the belief that lack of time for counseling and referral is a barrier to smoking cessation in cancer patients. Results showed that working as a physician, believing that active smoking or tobacco use affects cancer treatment outcomes, and working in a curative setting is associated with a higher likelihood of perceiving lack of time for counseling as a barrier to smoking cessation in patients. But in fact, this is where oncology staff felt the greatest need to implement smoking cessation, and so they see lack of time as a major barrier to not doing so.

All results of this study demonstrate the need for continued improvement in educating staff on how to advise, motivate, and support cancer patients in quitting smoking after a cancer diagnosis. Patients often have misconceptions [25] and are unaware of the consequences of continuing to smoke after a cancer diagnosis [26, 27]. Healthcare staff and patient education are critical and should be systematically integrated into oncology care. In 2012, Brach et al. noted that it is the duty of every health care organization, such as every cancer center, to ensure that patients have access to understandable health information. This should also include education about the negative consequences of continuing to smoke, after a diagnosis, as well as routinely asking cancer patients about their current and past smoking status. Recent studies [28] also show that patients are very interested and want to be informed and educated. This opportunity should be seized to ensure that cancer patients are well informed and know exactly where and how to get appropriate help to quit smoking.

## Limitations

When considering the results of this study, several limitations must also be taken into account. The present study is a cross-sectional study; therefore, associations cannot be interpreted in a causal manner [29].

In addition, all data were self-reported and were not supplemented by observations.

Another limitation to consider is that, although participants were recruited from our network of one local comprehensive cancer center, we are not able to track their exact job position and location. Therefore, there may be some bias in the selection of participants. It can also be discussed whether the participants are representative of the overall sample of oncology staff. Unfortunately, it is not possible to determine exactly who participated in the end; only the first step of contacting the leading oncology staff for further promotion and display of flyers in all wards and outpatient clinics in the network was standardized. Also, non-respondents cannot be analyzed, so the response rate cannot be calculated. This is due to the requirements of the clinics, which only allowed us to conduct this study on a completely anonymous basis to avoid social desirability [30] or shame effects [31], especially regarding the potential taboo topic of smoking as an oncology staff member.

To prevent participants from simply skipping questions if the suggested setting did not apply to them, we also added the pre-defined response option “setting does not apply.” However, this may have increased the number of participants who indicated that the setting did not apply when, maybe, they simply did not feel that the proposed behavior was important or had not yet engaged in this behavior. And this may have led us to overestimate the frequency of positive responses, so that the positive interactions with patients turn out to be lower.

In future studies, a prospective longitudinal approach to the topic analyzed would be of interest in order to be able to make stronger causal statements for possible predictor variables, as well as finding better ways to validate the reported practice patterns of oncology staff, e.g., through the corresponding analysis of patient-observed data.

## Conclusion

The results clearly show that the significance of assessing smoking status has already arrived in routine oncology care, but that the relevance of smoking cessation for cancer patients is rarely addressed. In the long term, a systematic approach is needed to determine the current and past smoking status of newly diagnosed cancer patient and to motivate currently smoking cancer patients to quit smoking. More structured referral to smoking intervention services is also needed. Much work needs to be done to better target and train oncology staff to address smoking cessation with cancer patients in a more systematic and professionally guided manner. A national program with finical resources to recruit and motivate patients and offer cessation would be a promising strategy.

## Supplementary Information

Below is the link to the electronic supplementary material.

Supplementary file1 (DOCX 18 KB)

Supplementary file2 (DOCX 16 KB)

Supplementary file3 (DOCX 17 KB)

Supplementary file4 (DOCX 55 KB)

Supplementary file5 (DOCX 54 KB)

## Data Availability

All relevant data are included in this publication. Detailed data will gladly be made available upon demand, e.g., for systematic reviews or meta-analysis.

## Abbreviations

*CI*: Confidence interval
*IQOS*: “I quit ordinary smoking”
*LPEK*: Local psychological Ethic committee at the center of psychosocial medicine Hamburg, Germany
*M*: Mean
*MD*: Medical doctor
*PASS*: Power Analysis & Sample Size
*SD*: Standard deviation
*SPSS*: Statistical Package for Social Sciences
