# Supplementary material for: Practice patterns, experiences, and challenges of German oncology health care staff with smoking cessation in patients with cancer: a cross-sectional survey study
Source: J Cancer Surviv. 2023 Nov 28;19(2):701–12. doi: 10.1007/s11764-023-01501-2 (PMC11926055; doi:10.1007/s11764-023-01501-2)
Supplement: Supplementary file 1 — Supplementary file1 (DOCX 18 KB) [file 11764_2023_1501_MOESM1_ESM.docx]

Table S1: Communication with patients on smoking^a^

|  | **Mean (SD) ^b^** | **CI [95 %]** | | | **d^c^** |
| --- | --- | --- | --- | --- | --- |
| 1. I ask my patients if they currently smoke cigarettes or use tobacco products (smoking status) |  |  |  |  |  |
| curative | 2.27 (1.59) | [2.11 | ; | 2.44] |  |
| palliative | 2.90 (1.83) | [2.71 | ; | 3.09] | -0.37 |
| 2. I ask patients if they use other tobacco products such as cigars, pipes, snuff, hookah/shisha, IQOS, etc. |  |  |  |  |  |
| curative | 3.55 (1.69) | [3.38 | ; | 3.73] |  |
| palliative | 3.90 (1.66) | [3.73 | ; | 4.07] | -0.21 |
| 3. I ask patients if they use electronic cigarettes or other electronic nicotine delivery devices |  |  |  |  |  |
| curative | 3.85 (1.55) | [3.69 | ; | 4.01] |  |
| palliative | 4.15 (1.47) | [4.00 | ; | 4.31] | -0.20 |
| 4. I ask patients if they have smoked in the past |  |  |  |  |  |
| curative | 2.53 (1.65) | [2.36 | ; | 2.70] |  |
| palliative | 3.03 (1.81) | [2.84 | ; | 3.21] | -0.29 |
| 5. When asking patients about tobacco use, I use a structured questionnaire or another structured method for asking questions |  |  |  |  |  |
| curative | 4.56 (1.53) | [4.40 | ; | 4.72] |  |
| palliative | 4.71 (1.44) | [4.56 | ; | 4.86] | -0.10 |
| 6. I ask patients who smoke or use tobacco if they want to quit smoking |  |  |  |  |  |
| curative | 3.16 (1.59) | [3.00 | ; | 3.33] |  |
| palliative | 3.74 (1.63) | [3.57 | ; | 3.91] | -0.36 |
| 7. I advise patients who smoke or use tobacco products to stop smoking |  |  |  |  |  |
| curative | 2.68 (1.60) | [2.52 | ; | 2.85] |  |
| palliative | 3.55 (1.69) | [3.37 | ; | 3.72] | -0.53 |
| 8. I discuss medication options such as nicotine replacement, bupropion, varenicline, etc. |  |  |  |  |  |
| curative | 3.84 (1.52) | [3.69 | ; | 4.00] |  |
| palliative | 4.22 (1.46) | [4.07 | ; | 4.38] | -0.26 |
| 9. I actively treat or refer patients for a smoking/tobacco cessation intervention |  |  |  |  |  |
| curative | 4.78 (1.20) | [4.65 | ; | 4.90] |  |
| palliative | 4.99 (1.06) | [4.88 | ; | 5.10] | -0.19 |
| 10. During follow-up appointments, I continue to assess smoking behavior in active smokers, and ask patients that have quit whether they might have relapsed back into tobacco use |  |  |  |  |  |
| curative | 3.97 (1.75) | [3.78 | ; | 4.15] |  |
| palliative | 4.49 (1.59) | [4.32 | ; | 4.65] | -0.31 |
|  |  |  |  |  |  |
| ^a^ Response options: 1 = always, 2 = most of the time, 3 = some of the time, 4 = rarely, 5 = never  ^b^ N = 354; ^c^ Cohen’s d of the differences in means between the two settings | | | |  |  |

**Submission information:**

**Article title:**

Practice patterns, experiences, and challenges of oncology health care professionals with smoking cessation in patients with cancer: taking a closer look

**Journal name:** Journal of Cancer Survivorship

**Author names:** Frederike Bokemeyer, Lisa Lebherz, Carsten Bokemeyer, Jeroen W.G. Derksen, Holger Schulz, Christiane Bleich

**Affiliation and e-mail address of the corresponding author:** Frederike Bokemeyer [f.bokemeyer@uke.de](mailto:f.bokemeyer@uke.de),

1. Department of Medical Psychology, University Medical Center Hamburg Eppendorf, Martinistraße 52, 20246 Hamburg, Germany

2. Center for Oncology, II. Medical Clinic and Polyclinic, University Medical Center Hamburg Eppendorf, Martinistraße 52, 20246 Hamburg, Germany
