# Supplementary material for: Practice patterns, experiences, and challenges of German oncology health care staff with smoking cessation in patients with cancer: a cross-sectional survey study
Source: J Cancer Surviv. 2023 Nov 28;19(2):701–12. doi: 10.1007/s11764-023-01501-2 (PMC11926055; doi:10.1007/s11764-023-01501-2)
Supplement: Supplementary file 2 — Supplementary file2 (DOCX 16 KB) [file 11764_2023_1501_MOESM2_ESM.docx]

Table S2: Perception of continued smoking after a cancer diagnosis

| Statement^a^ | Mean (SD) | CI [95 %] | | | N^b^ | d^c^ |
| --- | --- | --- | --- | --- | --- | --- |
| 1. Current smoking or tobacco use impacts treatment outcomes in cancer patients |  |  |  |  |  |  |
| curative | 1.84(0.85) | [1.74 | ; | 1.93] | 336 |  |
| palliative | 2.48(1.18) | [2.35 | ; | 2.61] | 323 | -0.63 |
| 2. Smoking/tobacco cessation should be a standard part of cancer treatment |  |  |  |  |  |  |
| curative | 1.82(0.96) | [1.72 | ; | 1.92] | 344 |  |
| palliative | 2.87(1.32) | [2.73 | ; | 3.02] | 335 | -0.92 |
|  |  |  |  |  |  |  |
| 3. Oncological professionals should receive more training on smoking and smoking cessation interventions (both settings combined) | 2.06(1.12) | [1.94 | ; | 2.18] | 354 |  |
|  |  |  |  |  |  |  |
| 4. I have had adequate training in smoking/tobacco cessation interventions (both settings combined) | 4.40(0.99) | [4.30 | ; | 4.50] | 354 |  |

| ^a^ Response options: I agree 1 = completely, 2 = mostly, 3 = somewhat, 4 = a little bit, 5 = not at all  ^b^ total N = 354; the remaining participants checked “not applicable”; ^b^ Cohen’s d of the differences in means between the two settings  **Submission information:**  **Article title:**  Practice patterns, experiences, and challenges of oncology health care professionals with smoking cessation in patients with cancer: taking a closer look  **Journal name:** Journal of Cancer Survivorship  **Author names:** Frederike Bokemeyer, Lisa Lebherz, Carsten Bokemeyer, Jeroen W.G. Derksen, Holger Schulz, Christiane Bleich  **Affiliation and e-mail address of the corresponding author:** Frederike Bokemeyer [f.bokemeyer@uke.de](mailto:f.bokemeyer@uke.de),  1. Department of Medical Psychology, University Medical Center Hamburg Eppendorf, Martinistraße 52, 20246 Hamburg, Germany  2. Center for Oncology, II. Medical Clinic and Polyclinic, University Medical Center Hamburg Eppendorf, Martinistraße 52, 20246 Hamburg, Germany |
| --- |
