# Supplementary material for: Practice patterns, experiences, and challenges of German oncology health care staff with smoking cessation in patients with cancer: a cross-sectional survey study
Source: J Cancer Surviv. 2023 Nov 28;19(2):701–12. doi: 10.1007/s11764-023-01501-2 (PMC11926055; doi:10.1007/s11764-023-01501-2)
Supplement: Supplementary file 3 — Supplementary file3 (DOCX 17 KB) [file 11764_2023_1501_MOESM3_ESM.docx]

Table S3: Potential barriers to smoking/tobacco cessation support

| **Potential barrier** | **Mean (SD)** | **CI [95 %]** | | | **N^a^** | **d^b^** |
| --- | --- | --- | --- | --- | --- | --- |
|  |  |  |  |  |  |  |
| The inability to get patients to quit smoking/tobacco use |  |  |  |  |  |  |
| curative | 2.67(1.12) | [2.55 | ; | 2.79] | 338 |  |
| palliative | 2.81(1.22) | [2.68 | ; | 2.95] | 319 | -0.12 |
| My own hesitation: it feels like bothering the patient, and I do not feel comfortable taking something away they might enjoy doing |  |  |  |  |  |  |
| curative | 3.27(1.23) | [3.14 | ; | 3.4] | 341 |  |
| palliative | 2.82(1.33) | [2.68 | ; | 2.96] | 332 | 0.35 |
| Waste of time; cessation after diagnosis does not affect outcomes in cancer patients |  |  |  |  |  |  |
| curative | 4.43(0.93) | [4.33 | ; | 4.54] | 327 |  |
| palliative | 3.91(1.21) | [3.78 | ; | 4.04] | 319 | 0.49 |
| Lack of time for counselling or to set up a referral |  |  |  |  |  |  |
| curative | 2.52(1.27) | [2.39 | ; | 2.66] | 337 |  |
| palliative | 2.73(1.29) | [2.59 | ; | 2.88] | 323 | -0.16 |
| No or limited provider reimbursement (financial reasons) |  |  |  |  |  |  |
| curative | 3.07(1.31) | [2.92 | ; | 3.22] | 299 |  |
| palliative | 3.14(1.27) | [2.99 | ; | 3.29] | 289 | -0.05 |
| Patient’s resistance to a cessation treatment |  |  |  |  |  |  |
| curative | 2.55(1.02) | [2.44 | ; | 2.66] | 340 |  |
| palliative | 2.49(1.06) | [2.37 | ; | 2.6] | 327 | 0.06 |
| Lack of training or experience in cessation interventions |  |  |  |  |  |  |
| curative | 2.11(1.08) | [1.99 | ; | 2.22] | 340 |  |
| palliative | 2.18(1.12) | [2.06 | ; | 2.3] | 328 | -0.06 |
| Lack of available resources or referrals for cessation interventions |  |  |  |  |  |  |
| curative | 2.02(1.06) | [1.9 | ; | 2.13] | 336 |  |
| palliative | 2.09(1.10) | [1.97 | ; | 2.21] | 326 | -0.06 |
| ^a^ Response options: I agree 1 = completely, 2 = mostly, 3 = somewhat, 4 = a little bit, 5 = not at all  ^a^ total N = 354; for smaller numbers of cases per question, the option "not applicable" was selected; ^b^ Cohen’s d of the differences in means between the two settings | | | | | | |

**Submission information:**

**Article title:**

Practice patterns, experiences, and challenges of oncology health care professionals with smoking cessation in patients with cancer: taking a closer look

**Journal name:** Journal of Cancer Survivorship

**Author names:** Frederike Bokemeyer, Lisa Lebherz, Carsten Bokemeyer, Jeroen W.G. Derksen, Holger Schulz, Christiane Bleich

**Affiliation and e-mail address of the corresponding author:** Frederike Bokemeyer [f.bokemeyer@uke.de](mailto:f.bokemeyer@uke.de),

1. Department of Medical Psychology, University Medical Center Hamburg Eppendorf, Martinistraße 52, 20246 Hamburg, Germany

2. Center for Oncology, II. Medical Clinic and Polyclinic, University Medical Center Hamburg Eppendorf, Martinistraße 52, 20246 Hamburg, Germany
