# Supplementary material for: Practice patterns, experiences, and challenges of German oncology health care staff with smoking cessation in patients with cancer: a cross-sectional survey study
Source: J Cancer Surviv. 2023 Nov 28;19(2):701–12. doi: 10.1007/s11764-023-01501-2 (PMC11926055; doi:10.1007/s11764-023-01501-2)
Supplement: Supplementary file 4 — Supplementary file4 (DOCX 55 KB) [file 11764_2023_1501_MOESM4_ESM.docx]

| **A. General information** | | | | | | | | | | | | | | |
| --- | --- | --- | --- | --- | --- | --- | --- | --- | --- | --- | --- | --- | --- | --- |
| 1. | Age | | | ⬜ Under 40 years | | | | | | | | | | |
|  |  |  |  | ⬜ 41-49 Years | | | | | | | | | | |
|  |  |  |  | ⬜ Over 50 years | | | | | | | | | | |
| 2. | Sex | | | ⬜ female | | | | | | ⬜ male | | | | ⬜ other |
| 3. | Which professional group do you belong to? | | | | | | | | | | | | | |
|  | ⬜ | | Physician | | | | | | | | | | | |
|  | ⬜ | | Scientist | | | | | | | | | | | |
|  | ⬜ | | Health nurse | | | | | | | | | | | |
|  | ⬜ | | Psychologist | | | | | | | | | | | |
|  | ⬜ | | Other (nutritional therapist, physiotherapist, surgical assistant, study nurse, chaplain, social worker, medical assistant) | | | | | | | | | | | |
| 4. | What is your primary area of clinical practice? | | | | | | | | | | | | | |
|  | ⬜ | Medical oncology | | | | | ⬜ | | Radiation oncology | | | | | |
|  | ⬜ | Surgical oncology | | | | | ⬜ | | Other: 🖉_______________________ | | | | | |
| 5. | What is your work setting? | | | | | | | | | | | | | |
|  | ⬜ | University or academic hospital | | | | | | ⬜ | | | Practice | | | |
|  | ⬜ | Hospital | | | | | | ⬜ | | | Other: 🖉_____________________ | | | |
| 6. | Have you ever smoked cigarettes/ tobacco in your life? | | | | | | | | | | | | | |
|  | ⬜ | No, never | | | ⬜ | Yes, but less than 100 | | | | | | ⬜ | Yes, more than 100 | |
| 7. | Do you currently smoke cigarettes/ tobacco? | | | | | | | | | | | | | |
|  | ⬜ | No, not at all | | | ⬜ | Yes, some days | | | | | | ⬜ | Yes, every day | |

| **B. Oncological treatment focuses** | | | | | | |
| --- | --- | --- | --- | --- | --- | --- |
| 1. | Which primary tumor types do you see most often? (max of 3 answers) | | | | | |
|  | ⬜ | Breast | ⬜ | Genitourinary | ⬜ | Skin |
|  | ⬜ | Lung | ⬜ | Head and neck | ⬜ | Brain |
|  | ⬜ | Gastrointestinal | ⬜ | Gynecologic | ⬜ | Other: |
|  | ⬜ | Lymphoma | ⬜ | Leukemia |  | 🖉_____________________ |

| **C. Physician’s interactions with patients** | | | | | | | | | |
| --- | --- | --- | --- | --- | --- | --- | --- | --- | --- |
| 1. | I ask patients if they currently smoke or use tobacco products. | | | | | | | | |
|  | Setting: | Always | Most of the time | | Some of the time | Rarely | Never | | N/A |
|  | Curative | ⬜ | ⬜ | | ⬜ | ⬜ | ⬜ | | ⬜ |
|  | Palliative | ⬜ | ⬜ | | ⬜ | ⬜ | ⬜ | | ⬜ |
| 2. | I ask patients if they use other tobacco products such as cigars, pipes, snuff, hookah/shisha, IQOS, etc. | | | | | | | | |
|  | Setting: | Always | Most of the time | | Some of the time | Rarely | Never | | N/A |
|  | Curative | ⬜ | ⬜ | | ⬜ | ⬜ | ⬜ | | ⬜ |
|  | Palliative | ⬜ | ⬜ | | ⬜ | ⬜ | ⬜ | | ⬜ |
| 3. | I ask patients if they use electronic cigarettes or other electronic nicotine delivery devices. | | | | | | | | |
|  | Setting: | Always | Most of the time | | Some of the time | Rarely | Never | | N/A |
|  | Curative | ⬜ | ⬜ | | ⬜ | ⬜ | ⬜ | | ⬜ |
|  | Palliative | ⬜ | ⬜ | | ⬜ | ⬜ | ⬜ | | ⬜ |
| 4. | I ask my patients if they have smoked in the past. | | | | | | | | |
|  | Setting: | Always | Most of the time | | Some of the time | Rarely | Never | | N/A |
|  | Curative | ⬜ | ⬜ | | ⬜ | ⬜ | ⬜ | | ⬜ |
|  | Palliative | ⬜ | ⬜ | | ⬜ | ⬜ | ⬜ | | ⬜ |
| 5. | When asking about tobacco use, I use a structured questionnaire or other structured method for asking questions. | | | | | | | | |
|  | Setting: | Always | Most of the time | | Some of the time | Rarely | Never | | N/A |
|  | Curative | ⬜ | ⬜ | | ⬜ | ⬜ | ⬜ | | ⬜ |
|  | Palliative | ⬜ | ⬜ | | ⬜ | ⬜ | ⬜ | | ⬜ |
| 6. | I ask patients who smoke or use tobacco if they want to quit smoking. | | | | | | | | |
|  | Setting: | Always | Most of the time | | Some of the time | Rarely | Never | | N/A |
|  | Curative | ⬜ | ⬜ | | ⬜ | ⬜ | ⬜ | | ⬜ |
|  | Palliative | ⬜ | ⬜ | | ⬜ | ⬜ | ⬜ | | ⬜ |
| 7. | I advise patients who smoke or use tobacco products to stop smoking. | | | | | | | | |
|  | Setting: | Always | Most of the time | | Some of the time | Rarely | Never | | N/A |
|  | Curative | ⬜ | ⬜ | | ⬜ | ⬜ | ⬜ | | ⬜ |
|  | Palliative | ⬜ | ⬜ | | ⬜ | ⬜ | ⬜ | | ⬜ |
| 8. | I discuss medication options such as nicotine replacement, bupropion, varenicline, etc. | | | | | | | | |
|  | Setting: | Always | Most of the time | | Some of the time | Rarely | Never | | N/A |
|  | Curative | ⬜ | ⬜ | | ⬜ | ⬜ | ⬜ | | ⬜ |
|  | Palliative | ⬜ | ⬜ | | ⬜ | ⬜ | ⬜ | | ⬜ |
| 9. | I actively treat or refer patients for a smoking/ tobacco cessation intervention. | | | | | | | | |
|  | Setting: | Always | Most of the time | | Some of the time | Rarely | Never | | N/A |
|  | Curative | ⬜ | ⬜ | | ⬜ | ⬜ | ⬜ | | ⬜ |
|  | Palliative | ⬜ | ⬜ | | ⬜ | ⬜ | ⬜ | | ⬜ |
| 10. | During follow-up appointments, I continue to assess smoking behavior in active smokers, and ask patients that have quit whether they might have relapsed back into tobacco use. | | | | | | | | |
|  | Setting: | Always | Most of the time | | Some of the time | Rarely | Never | | N/A |
|  | Curative | ⬜ | ⬜ | | ⬜ | ⬜ | ⬜ | | ⬜ |
|  | Palliative | ⬜ | ⬜ | | ⬜ | ⬜ | ⬜ | | ⬜ |
| 11. | My interactions with patients regarding smoking/ tobacco use differ between tobacco-related vs. non tobacco-related cancers. | | | | | | | | |
|  | Setting: | No | | Yes, I mostly discuss this with patients with **tobacco-related** cancers | | Yes, I mostly discuss this with patients with **non tobacco-related** cancers | | N/A | |
|  | Curative | ⬜ | | ⬜ | | ⬜ | | ⬜ | |
|  | Palliative | ⬜ | | ⬜ | | ⬜ | | ⬜ | |

| **D. Physician’s perceptions of tobacco use in patients with cancer:** | | | | | | |
| --- | --- | --- | --- | --- | --- | --- |
| 1. | Current smoking or tobacco use impacts treatment outcomes in cancer patients. | | | | | |
|  | Setting: | Strongly agree | Agree | No opinion or neutral | Disagree | Strongly disagree |
|  | Curative | ⬜ | ⬜ | ⬜ | ⬜ | ⬜ |
|  | Palliative | ⬜ | ⬜ | ⬜ | ⬜ | ⬜ |
| 2. | Smoking/tobacco cessation should be a standard part of cancer treatment interventions. | | | | | |
|  | Setting: | Strongly agree | Agree | No opinion or neutral | Disagree | Strongly disagree |
|  | Curative | ⬜ | ⬜ | ⬜ | ⬜ | ⬜ |
|  | Palliative | ⬜ | ⬜ | ⬜ | ⬜ | ⬜ |

| 3. | I have had adequate training in smoking/tobacco cessation interventions. | | | | | | | | |
| --- | --- | --- | --- | --- | --- | --- | --- | --- | --- |
|  |  | | Strongly agree | Agree | | | No opinion or neutral | Disagree | Strongly disagree |
|  |  | | ⬜ | ⬜ | | | ⬜ | ⬜ | ⬜ |
| 4. | Clinicians need more training in smoking/tobacco assessment and cessation interventions. | | | | | | | | |
|  |  | | Strongly agree | Agree | | | No opinion or neutral | Disagree | Strongly disagree |
|  |  | | ⬜ | ⬜ | | | ⬜ | ⬜ | ⬜ |
| 5. | Which of the following providers do you think is appropriate to provide **cessation** support for cancer patients on a regular basis?  (check at least one) | | | | | | | | |
|  | ⬜ | General practitioner | | | ⬜ | Clinical support staff within the clinic such as a psychologist, or social worker | | | |
|  | ⬜ | Specialists (other than treating oncologists) | | | ⬜ | The treating oncologist | | | |
|  | ⬜ | Mid-level clinician such as a nurse practitioner or physician assistant | | | ⬜ | I would not use any of the above resources | | | |
|  | ⬜ | Other: 🖉_____________________________ | | | | | | | |
| 6. | What type of dedicated smoking/tobacco cessation program does your Facility/ Practice have available for your cancer patients (check at least one) | | | | | | | | |
|  | ⬜ | A tobacco cessation clinic/specialist that provides face-to-face counseling | | | ⬜ | Provision of tobacco cessation materials (such as pamphlets, websites, or a DVD) | | | |
|  | ⬜ | A tobacco cessation specialist who provides telephone-based counseling | | | ⬜ | None to my knowledge | | | |
|  |  |  |  |  | ⬜ | I don’t know | | | |
|  | ⬜ | A tobacco cessation clinic/specialist that provides pharmacotherapy | | | ⬜ | Other: 🖉_____________________ | | | |

| **E. Potential barriers to smoking/tobacco cessation interventions:** | | | | | | | |
| --- | --- | --- | --- | --- | --- | --- | --- |
| 1. | The inability to get patients to quit smoking/tobacco use. | | | | | | |
|  | Setting: | Strongly agree | Agree | No opinion or neutral | Disagree | Strongly disagree | N/A |
|  | Curative | ⬜ | ⬜ | ⬜ | ⬜ | ⬜ | ⬜ |
|  | Palliative | ⬜ | ⬜ | ⬜ | ⬜ | ⬜ | ⬜ |

| 2. | My own hesitation; it feels like bothering the patient, and I do not feel comfortable taking something away they might enjoy doing. | | | | | | |
| --- | --- | --- | --- | --- | --- | --- | --- |
|  | Setting: | Strongly agree | Agree | No opinion or neutral | Disagree | Strongly disagree | N/A |
|  | Curative | ⬜ | ⬜ | ⬜ | ⬜ | ⬜ | ⬜ |
|  | Palliative | ⬜ | ⬜ | ⬜ | ⬜ | ⬜ | ⬜ |
| 3. | Waste of time; cessation after diagnosis does not affect outcomes in cancer patients. | | | | | | |
|  | Setting: | Strongly agree | Agree | No opinion or neutral | Disagree | Strongly disagree | N/A |
|  | Curative | ⬜ | ⬜ | ⬜ | ⬜ | ⬜ | ⬜ |
|  | Palliative | ⬜ | ⬜ | ⬜ | ⬜ | ⬜ | ⬜ |
| 4. | Lack of time for counseling or to set up a referral. | | | | | | |
|  | Setting: | Strongly agree | Agree | No opinion or neutral | Disagree | Strongly disagree | N/A |
|  | Curative | ⬜ | ⬜ | ⬜ | ⬜ | ⬜ | ⬜ |
|  | Palliative | ⬜ | ⬜ | ⬜ | ⬜ | ⬜ | ⬜ |
| 5. | No or limited provider reimbursement (financial reasons). | | | | | | |
|  | Setting: | Strongly agree | Agree | No opinion or neutral | Disagree | Strongly disagree | N/A |
|  | Curative | ⬜ | ⬜ | ⬜ | ⬜ | ⬜ | ⬜ |
|  | Palliative | ⬜ | ⬜ | ⬜ | ⬜ | ⬜ | ⬜ |
| 6. | Patient’s resistance to a cessation treatment. | | | | | | |
|  | Setting: | Strongly agree | Agree | No opinion or neutral | Disagree | Strongly disagree | N/A |
|  | Curative | ⬜ | ⬜ | ⬜ | ⬜ | ⬜ | ⬜ |
|  | Palliative | ⬜ | ⬜ | ⬜ | ⬜ | ⬜ | ⬜ |
| 7. | Lack of training or experience in cessation interventions. | | | | | | |
|  | Setting: | Strongly agree | Agree | No opinion or neutral | Disagree | Strongly disagree | N/A |
|  | Curative | ⬜ | ⬜ | ⬜ | ⬜ | ⬜ | ⬜ |
|  | Palliative | ⬜ | ⬜ | ⬜ | ⬜ | ⬜ | ⬜ |
| 8. | Lack of available resources or referrals for cessation interventions. | | | | | | |
|  | Setting: | Strongly agree | Agree | No opinion or neutral | Disagree | Strongly disagree | N/A |
|  | Curative | ⬜ | ⬜ | ⬜ | ⬜ | ⬜ | ⬜ |
|  | Palliative  End. We thank you very much for your participation. | ⬜ | ⬜ | ⬜ | ⬜ | ⬜ | ⬜ |

**Submission information:**

**Article title:**

Practice patterns, experiences, and challenges of oncology health care professionals with smoking cessation in patients with cancer: taking a closer look

**Journal name:** Journal of Cancer Survivorship

**Author names:** Frederike Bokemeyer, Lisa Lebherz, Carsten Bokemeyer, Jeroen W.G. Derksen, Holger Schulz, Christiane Bleich

**Affiliation and e-mail address of the corresponding author:** Frederike Bokemeyer [f.bokemeyer@uke.de](mailto:f.bokemeyer@uke.de),

1. Department of Medical Psychology, University Medical Center Hamburg Eppendorf, Martinistraße 52, 20246 Hamburg, Germany

2. Center for Oncology, II. Medical Clinic and Polyclinic, University Medical Center Hamburg Eppendorf, Martinistraße 52, 20246 Hamburg, Germany
