# Supplementary material for: Practice patterns, experiences, and challenges of German oncology health care staff with smoking cessation in patients with cancer: a cross-sectional survey study
Source: J Cancer Surviv. 2023 Nov 28;19(2):701–12. doi: 10.1007/s11764-023-01501-2 (PMC11926055; doi:10.1007/s11764-023-01501-2)
Supplement: Supplementary file 5 — Supplementary file5 (DOCX 54 KB) [file 11764_2023_1501_MOESM5_ESM.docx]

| **A. Allgemeine Angaben** | | | | | | | | | | | | | | |
| --- | --- | --- | --- | --- | --- | --- | --- | --- | --- | --- | --- | --- | --- | --- |
| 1. | Alter | | | ⬜ Unter 40 Jahren | | | | | | | | | | |
|  |  |  |  | ⬜ 41-49 Jahre | | | | | | | | | | |
|  |  |  |  | ⬜ Über 50 Jahre | | | | | | | | | | |
| 2. | Geschlecht | | | ⬜ weiblich | | | | | | ⬜ männlich | | | | ⬜ divers |
| 3. | Welcher Berufsgruppe gehören Sie an? | | | | | | | | | | | | | |
|  | ⬜ | | Arzt/Ärztin | | | | | | | | | | | |
|  | ⬜ | | Wissenschaftler:in | | | | | | | | | | | |
|  | ⬜ | | Gesundheits-/Krankenpfleger:in | | | | | | | | | | | |
|  | ⬜ | | Psycholog:in | | | | | | | | | | | |
|  | ⬜ | | Sonstige ((Ernährungstherapeut:in, Physiotherapeut:in, Operationstechnische Assistenz, Study Nurse, Seelsorger:in, Sozialarbeiter:in, Med. Fachangestellter:in) | | | | | | | | | | | |
| 4. | Welches ist Ihr primäres Gebiet der klinischen Praxis? | | | | | | | | | | | | | |
|  | ⬜ | Medizinische Onkologie | | | | | ⬜ | | Onkologische Strahlentherapie | | | | | |
|  | ⬜ | Operative Onkologie | | | | | ⬜ | | Sonstiges: 🖉_______________________ | | | | | |
| 5. | Wo arbeiten Sie aktuell? | | | | | | | | | | | | | |
|  | ⬜ | Universitäts- oder Lehrkrankenhaus | | | | | | ⬜ | | | Praxis | | | |
|  | ⬜ | Krankenhaus | | | | | | ⬜ | | | Sonstiges: 🖉_____________________ | | | |
| 6. | Haben Sie jemals in Ihrem Leben Zigaretten oder andere Tabakprodukte geraucht? | | | | | | | | | | | | | |
|  | ⬜ | Nie | | | ⬜ | Ja, unter 100 | | | | | | ⬜ | Ja, mehr als 100 | |
| 7. | Rauchen Sie selbst aktuell Zigaretten oder andere Tabakprodukte? | | | | | | | | | | | | | |
|  | ⬜ | Nein | | | ⬜ | Ja, manchmal | | | | | | ⬜ | Ja, jeden Tag | |

| **B. Onkologische Behandlungsschwerpunkte** | | | | | | |
| --- | --- | --- | --- | --- | --- | --- |
| 1. | Welche Primärtumorarten behandeln Sie am häufigsten oder an welchen Behandlungen sind Sie am häufigsten beteiligt? (max. 3 Nennungen) | | | | | |
|  | ⬜ | Brusttumor | ⬜ | Urogenitaler Tumor | ⬜ | Hauttumor |
|  | ⬜ | Lungentumor | ⬜ | Kopf- und Hals -Tumor | ⬜ | Hirntumor |
|  | ⬜ | Gastrointestinaler Tumor | ⬜ | Gynäkologischer Tumor (Ovar, Zervix) | ⬜ | Sonstiges: |
|  | ⬜ | Lymphom | ⬜ | Leukämie |  | 🖉_____________________ |

| **C. Interaktion mit den Patient:innen** | | | | | | | | | |
| --- | --- | --- | --- | --- | --- | --- | --- | --- | --- |
| 1. | Ich frage meine Patient:innen, ob sie aktuell Zigaretten rauchen | | | | | | | | |
|  | Setting: | immer | meistens | | manchmal | selten | nie | | Setting trifft  nicht zu |
|  | Kurativ | ⬜ | ⬜ | | ⬜ | ⬜ | ⬜ | | ⬜ |
|  | Palliativ | ⬜ | ⬜ | | ⬜ | ⬜ | ⬜ | | ⬜ |
| 2. | Ich frage meine Patient:innen, ob sie andere Tabakprodukte wie Zigarren, Pfeifen, Schnupftabak, Wasserpfeife/Shisha, IQOS o.ä. konsumieren. | | | | | | | | |
|  | Setting: | immer | meistens | | manchmal | selten | nie | | Setting trifft nicht zu |
|  | Kurativ | ⬜ | ⬜ | | ⬜ | ⬜ | ⬜ | | ⬜ |
|  | Palliativ | ⬜ | ⬜ | | ⬜ | ⬜ | ⬜ | | ⬜ |
| 3. | Ich frage meine Patient:innen, ob sie elektronische Zigaretten oder andere elektronische Nikotinabgabegeräte benutzen. | | | | | | | | |
|  | Setting: | immer | meistens | | manchmal | selten | nie | | Setting trifft  nicht zu |
|  | Kurativ | ⬜ | ⬜ | | ⬜ | ⬜ | ⬜ | | ⬜ |
|  | Palliativ | ⬜ | ⬜ | | ⬜ | ⬜ | ⬜ | | ⬜ |
| 4. | Ich frage meine Patient:innen, ob Sie in der Vergangenheit geraucht haben. | | | | | | | | |
|  | Setting: | immer | meistens | | manchmal | selten | nie | | Setting trifft  nicht zu |
|  | Kurativ | ⬜ | ⬜ | | ⬜ | ⬜ | ⬜ | | ⬜ |
|  | Palliativ | ⬜ | ⬜ | | ⬜ | ⬜ | ⬜ | | ⬜ |
| 5. | Wenn ich meine Patient:innen nach dem Tabakkonsum frage, verwende ich einen strukturierten Fragebogen oder eine andere strukturierte Methode, um Fragen zu stellen. | | | | | | | | |
|  | Setting: | immer | meistens | | manchmal | selten | nie | | Setting trifft  nicht zu |
|  | Kurativ | ⬜ | ⬜ | | ⬜ | ⬜ | ⬜ | | ⬜ |
|  | Palliativ | ⬜ | ⬜ | | ⬜ | ⬜ | ⬜ | | ⬜ |
| 6. | Ich frage meine Patient:innen, die rauchen bzw. Tabak konsumieren, ob sie mit dem Rauchen aufhören wollen. | | | | | | | | |
|  | Setting: | immer | meistens | | manchmal | selten | nie | | Setting trifft  nicht zu |
|  | Kurativ | ⬜ | ⬜ | | ⬜ | ⬜ | ⬜ | | ⬜ |
|  | Palliativ | ⬜ | ⬜ | | ⬜ | ⬜ | ⬜ | | ⬜ |
| 7. | Ich rate meinen Patient:innen, die rauchen oder Tabakprodukte konsumieren, mit dem Rauchen aufzuhören. | | | | | | | | |
|  | Setting: | immer | meistens | | manchmal | selten | nie | | Setting trifft  nicht zu |
|  | Kurativ | ⬜ | ⬜ | | ⬜ | ⬜ | ⬜ | | ⬜ |
|  | Palliativ | ⬜ | ⬜ | | ⬜ | ⬜ | ⬜ | | ⬜ |
| 8. | Ich bespreche Medikationsmöglichkeiten wie Nikotinersatz, Bupropion, Vareniclin usw. | | | | | | | | |
|  | Setting: | immer | meistens | | manchmal | selten | nie | | Setting trifft  nicht zu |
|  | Kurativ | ⬜ | ⬜ | | ⬜ | ⬜ | ⬜ | | ⬜ |
|  | Palliativ | ⬜ | ⬜ | | ⬜ | ⬜ | ⬜ | | ⬜ |
| 9. | Ich behandele oder überweise meine Patient:innen aktiv für eine Raucherentwöhnungsintervention. | | | | | | | | |
|  | Setting: | immer | meistens | | manchmal | selten | nie | | Setting trifft  nicht zu |
|  | Kurativ | ⬜ | ⬜ | | ⬜ | ⬜ | ⬜ | | ⬜ |
|  | Palliativ | ⬜ | ⬜ | | ⬜ | ⬜ | ⬜ | | ⬜ |
| 10. | Bei Nachsorgeterminen befrage ich aktive Raucher:innen nach ihrem aktuellen Rauchverhalten und frage Patient:innen, die mit dem Rauchen aufgehört haben, ob sie möglicherweise wieder rückfällig geworden sind und Tabak konsumieren. | | | | | | | | |
|  | Setting: | immer | meistens | | manchmal | selten | nie | | Setting trifft  nicht zu |
|  | Kurativ | ⬜ | ⬜ | | ⬜ | ⬜ | ⬜ | | ⬜ |
|  | Palliativ | ⬜ | ⬜ | | ⬜ | ⬜ | ⬜ | | ⬜ |
| 11. | In meinen Gesprächen mit Patient:innen bezüglich ihres Rauchens/Tabakkonsums unterscheide ich zwischen tabakbezogenen und nicht-tabakbezogenen Krebsarten. | | | | | | | | |
|  | Setting: | nein | | ja, bespreche ich meist bei **tabakassoziierten** Krebsarten | | ja, bespreche ich meist bei **nicht-tabakassoziierten** Krebsarten | | Setting trifft  nicht zu | |
|  | Kurativ | ⬜ | | ⬜ | | ⬜ | | ⬜ | |
|  | Palliativ | ⬜ | | ⬜ | | ⬜ | | ⬜ | |

| **D. Bewertung von Aussagen zum Tabakkonsum bei Patient:innen mit Krebs** | | | | | | |
| --- | --- | --- | --- | --- | --- | --- |
| 1. | Aktives Rauchen oder Tabakkonsum beeinflusst die Behandlungsergebnisse bei Krebspatient:innen | | | | | |
|  | Setting: | stimmt sehr | stimmt | mittelmäßig | stimmt nicht | stimmt überhaupt nicht |
|  | Kurativ | ⬜ | ⬜ | ⬜ | ⬜ | ⬜ |
|  | Palliativ | ⬜ | ⬜ | ⬜ | ⬜ | ⬜ |
| 2. | Raucher-/Tabakentwöhnung sollte ein Standardbestandteil der Krebsbehandlung sein. | | | | | |
|  | Setting: | stimmt sehr | stimmt | mittelmäßig | stimmt nicht | stimmt überhaupt nicht |
|  | Kurativ | ⬜ | ⬜ | ⬜ | ⬜ | ⬜ |
|  | Palliativ | ⬜ | ⬜ | ⬜ | ⬜ | ⬜ |

| 3. | Ich habe eine angemessene Schulung in Interventionen zur Raucherentwöhnung/ Tabakentwöhnung erhalten. | | | | | | | | |
| --- | --- | --- | --- | --- | --- | --- | --- | --- | --- |
|  |  | | stimmt sehr | stimmt | | | mittelmäßig | stimmt nicht | stimmt überhaupt nicht |
|  |  | | ⬜ | ⬜ | | | ⬜ | ⬜ | ⬜ |
| 4. | Medizinisches Personal braucht mehr Schulungen zu Interventionen bzgl. des Rauchens und der Raucherentwöhnung. | | | | | | | | |
|  |  | | stimmt sehr | stimmt | | | mittelmäßig | stimmt nicht | stimmt überhaupt nicht |
|  |  | | ⬜ | ⬜ | | | ⬜ | ⬜ | ⬜ |
| 5. | Welche der folgenden Anbieter:innen ist Ihrer Meinung nach geeignet, Krebspatient:innen regelmäßig Unterstützung bei der **Tabakentwöhnung** zu bieten?  (Mehrfachnenngen möglich) | | | | | | | | |
|  | ⬜ | Hausärzt:innen | | | ⬜ | Anderes klinisches Personal wie z.B. Psycholog:innen oder Sozialarbeiter:innen | | | |
|  | ⬜ | Fachärzt:innen (andere als behandelnden Onkolog:innen) | | | ⬜ | Behandelnde Onkolog:in | | | |
|  | ⬜ | Pflegepersonal oder medizinische Fachangestellte | | | ⬜ | Meiner Meinung nach ist keiner der Genannten dafür geeignet | | | |
|  | ⬜ | Andere: 🖉_____________________________ | | | | | | | |
| 6. | Welche Art von speziellem Raucher-/Tabakentwöhnungsprogramm bietet Ihre Einrichtung/Praxis Ihren Krebspatient:innen an? (Mehrfachnennungen möglich). | | | | | | | | |
|  | ⬜ | Spezialist:in für Tabakent-wöhnung, die/der persönliche Beratung anbietet. | | | ⬜ | Bereitstellung von Materialien zur Tabakentwöhnung, wie z.B. Broschüren/ Webseiten/Videos | | | |
|  | ⬜ | Spezialist:in für Tabak-entwöhnung, die/der telefonische Beratung anbietet. | | | ⬜ | Meines Wissens nach keine | | | |
|  |  |  |  |  | ⬜ | Ich weiß nicht | | | |
|  | ⬜ | Spezialist:in für Tabak-entwöhnung, die auch Pharmakotherapie anbietet. | | | ⬜ | Sonstiges: 🖉_____________________ | | | |

| **E. Mögliche Hindernisse bei der Raucher- / Tabakentwöhnung** | | | | | | | |
| --- | --- | --- | --- | --- | --- | --- | --- |
| 1. | Die Machtlosigkeit der Behandler:innen, den Patient:innen dazu zu bringen, mit dem Rauchen/Tabakkonsum aufzuhören. | | | | | | |
|  | Setting: | stimmt sehr | stimmt | mittel-mäßig | stimmt nicht | stimmt überhaupt nicht | Setting trifft  nicht zu |
|  | Kurativ | ⬜ | ⬜ | ⬜ | ⬜ | ⬜ | ⬜ |
|  | Palliativ | ⬜ | ⬜ | ⬜ | ⬜ | ⬜ | ⬜ |

| 2. | Zögern der Behandler:innen; es fühlt sich an, als würde man die Patient:innen belästigen, und man fühlt sich nicht wohl dabei, ihnen etwas wegzunehmen, was ihnen vielleicht Entlastung verschafft. | | | | | | |
| --- | --- | --- | --- | --- | --- | --- | --- |
|  | Setting: | stimmt sehr | stimmt | mittel-mäßig | stimmt nicht | stimmt überhaupt nicht | Setting trifft  nicht zu |
|  | Kurativ | ⬜ | ⬜ | ⬜ | ⬜ | ⬜ | ⬜ |
|  | Palliativ | ⬜ | ⬜ | ⬜ | ⬜ | ⬜ | ⬜ |
| 3. | Es ist Zeitverschwendung; ein Abbruch nach der Diagnose hat keinen Einfluss auf die Behandlungsergebnisse bei Krebspatient:innen. | | | | | | |
|  | Setting: | stimmt sehr | stimmt | mittel-mäßig | stimmt nicht | stimmt überhaupt nicht | Setting trifft  nicht zu |
|  | Kurativ | ⬜ | ⬜ | ⬜ | ⬜ | ⬜ | ⬜ |
|  | Palliativ | ⬜ | ⬜ | ⬜ | ⬜ | ⬜ | ⬜ |
| 4. | Mangelnde Zeit für eine entsprechende angemessene Beratung oder für eine Überweisung. | | | | | | |
|  | Setting: | stimmt sehr | stimmt | mittel-mäßig | stimmt nicht | stimmt überhaupt nicht | Setting trifft  nicht zu |
|  | Kurativ | ⬜ | ⬜ | ⬜ | ⬜ | ⬜ | ⬜ |
|  | Palliativ | ⬜ | ⬜ | ⬜ | ⬜ | ⬜ | ⬜ |
| 5. | Keine oder nur eingeschränkte Kostenerstattung für die Patient:innen (finanzielle Gründe). | | | | | | |
|  | Setting: | stimmt sehr | stimmt | mittel-mäßig | stimmt nicht | stimmt überhaupt nicht | Setting trifft  nicht zu |
|  | Kurativ | ⬜ | ⬜ | ⬜ | ⬜ | ⬜ | ⬜ |
|  | Palliativ | ⬜ | ⬜ | ⬜ | ⬜ | ⬜ | ⬜ |
| 6. | Widerstand der Patienten:innen gegen eine Raucherentwöhnung. | | | | | | |
|  | Setting: | stimmt sehr | stimmt | mittel-mäßig | stimmt nicht | stimmt überhaupt nicht | Setting trifft  nicht zu |
|  | Kurativ | ⬜ | ⬜ | ⬜ | ⬜ | ⬜ | ⬜ |
|  | Palliativ | ⬜ | ⬜ | ⬜ | ⬜ | ⬜ | ⬜ |
| 7. | Fehlende Schulung oder Erfahrung des Personals bezgl. Raucherentwöhnung. | | | | | | |
|  | Setting: | stimmt sehr | stimmt | mittel-mäßig | stimmt nicht | stimmt überhaupt nicht | Setting trifft  nicht zu |
|  | Kurativ | ⬜ | ⬜ | ⬜ | ⬜ | ⬜ | ⬜ |
|  | Palliativ | ⬜ | ⬜ | ⬜ | ⬜ | ⬜ | ⬜ |
| 8. | Mangel an oder fehlende Kenntnis von verfügbaren Angeboten oder Überweisungsmöglichkeiten für Entwöhnungsinterventionen. | | | | | | |
|  | Setting: | stimmt sehr | stimmt | mittel-mäßig | stimmt nicht | stimmt überhaupt nicht | Setting trifft  nicht zu |
|  | Kurativ | ⬜ | ⬜ | ⬜ | ⬜ | ⬜ | ⬜ |
|  | Palliativ  Ende. Wir bedanken uns herzlich für Ihre Teilnahme. | ⬜ | ⬜ | ⬜ | ⬜ | ⬜ | ⬜ |

**Submission information:**

**Article title:**

Practice patterns, experiences, and challenges of oncology health care professionals with smoking cessation in patients with cancer: taking a closer look

**Journal name:** Journal of Cancer Survivorship

**Author names:** Frederike Bokemeyer, Lisa Lebherz, Carsten Bokemeyer, Jeroen W.G. Derksen, Holger Schulz, Christiane Bleich

**Affiliation and e-mail address of the corresponding author:** Frederike Bokemeyer [f.bokemeyer@uke.de](mailto:f.bokemeyer@uke.de),

1. Department of Medical Psychology, University Medical Center Hamburg Eppendorf, Martinistraße 52, 20246 Hamburg, Germany

2. Center for Oncology, II. Medical Clinic and Polyclinic, University Medical Center Hamburg Eppendorf, Martinistraße 52, 20246 Hamburg, Germany
